# Diurnal Characteristics of Ecosystem Respiration of Alpine Meadow on the Qinghai-Tibetan Plateau: Implications for Carbon Budget Estimation

**DOI:** 10.1155/2013/289754

**Published:** 2013-06-24

**Authors:** Yu Qin, Shuhua Yi

**Affiliations:** State Key Laboratory of Cryospheric Sciences, Cold and Arid Regions Environmental and Engineering Research Institute, Chinese Academy of Sciences, 320 Donggang West Road, Lanzhou 730000, China

## Abstract

Accurately estimating daily mean ecosystem respiration rate (Re) is important for understanding how ecosystem carbon budgets will respond to climate change. Usually, daily mean Re is represented by measurement using static chamber on alpine meadow ecosystems from 9:00 to 11:00 h a.m. local time directly. In the present study, however, we found that the calculated daily mean Re from 9:00 to 11:00 h a.m. local time was significantly higher than that from 0:00 to 23:30 h local time in an alpine meadow site, which might be caused by special climate condition on the Qinghai-Tibetan Plateau. Our results indicated that the calculated daily mean Re from 9:00 to 11:00 h a.m. local time cannot be used to represent daily mean Re directly.

## 1. Introduction

Great concerns over global warming and climate change have been proposed for improving the accuracy in estimating carbon flux in terrestrial ecosystems [1–3]. Static chamber method has widely been applied to measure ecosystem respiration in different ecosystems [4–6]. This method requires manual operation, therefore, it is a common practice to measure respirations of a period in a day to represent daily mean value, for example, 06:00–11:00 h a.m. [7], 9:00–11:00 h a.m. [8], and 14:00–16:00 h p.m [9]. Re measured between 9:00 and 11:00 h a.m. local time has been used to represent the daily mean value for the alpine meadow ecosystems on the Qinghai-Tibetan Plateau [10–15] and other ecosystems [8, 16, 17]. However, it is unknown whether this method is valid on the Qinghai-Tibetan Plateau, which has unique climate condition [18]. Therefore, in this study, we carried out round-the-clock field observation of ecosystem respiration per half-hour using an automated soil CO_2_ flux system to test this method. 

The field measurement was carried out in an alpine meadow at 3,887 m a.s.l. in Shule River Basin at the southeast 45 km far away Suli county (98°18′33.2′′ E, 38°25′′13.5′′ N), the western part of Qilian Mountain, which is on the northeast edge of the Qinghai-Tibetan Plateau, Qinghai Province, China. The climate belongs to continental climate and is mainly controlled by westerly winds, with annual average precipitation being 200–400 mm, of which nearly 90% falls in the growing season (May–September), and the annual mean temperature ranged from −4.0 to −19.4°C [19]. Soils are classified as felty soils [20]. The study site, ~100 × 100 m^2^, has been fenced in 2010 to exclude the grazing activities of sheep and yaks. The dominant vegetations are *Kobresia capillifolia* and *Carex moorcroftii*. The permafrost type is transition according to the classified method by Cheng and Wang [21]. Three 2 × 2 m^2^ plots were set up randomly for the measurement of ecosystem respiration, and all the selected plots were expected to be less in spatial heterogeneity by visual inspection of the vegetation. Half-hour Re values were measured every 3 to 15 days depending on weather conditions during the whole growing season (May–September) in 2012 using an automated soil CO_2_ flux system (LI-8150, LI-COR Biosciences, Lincoln, NE, USA) equipped with LI-COR-8100-104 long-term chamber. Three polyvinyl chloride collars 20 cm in diameter and 12 cm in height were used for measurements. Collars were inserted into soil at 8-9 cm. To reduce a disturbance-induced CO_2_ efflux, all collars were installed 24 h prior to the first measurement. Soil temperature at 5 cm and moisture at 7 cm below soil surface were measured at each chamber simultaneously while Re was measured. To eliminate artifacts due to the Venturi effect [22], we excluded measured values of Re when wind speed exceeded 7.5 m s^−1^ according to Xu et al. [23]. Data of the half-hour wind speed was derived from the meteorological stations in our study site for the period of May to September, 2012. However, our exclusion can maintain the reliability of values because field observation supplied enough data for estimating daily mean Re.

**Figure 1:**
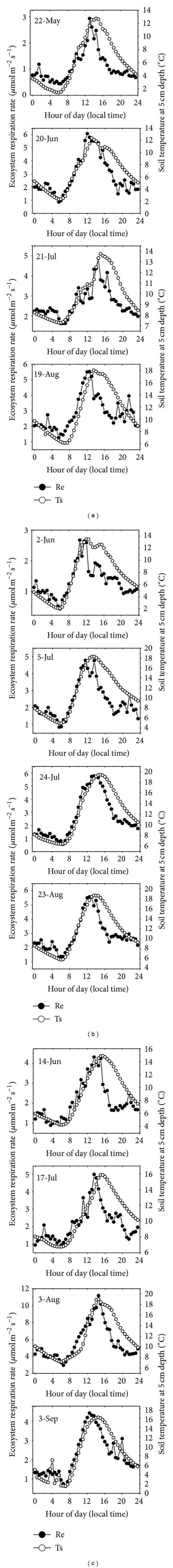
Diurnal variations of ecosystem respiration rate (*μ*moL m^−2^ s^−1^) and soil temperature at 5 cm depth (°C) during the grown season from May to September.

**Figure 2:**
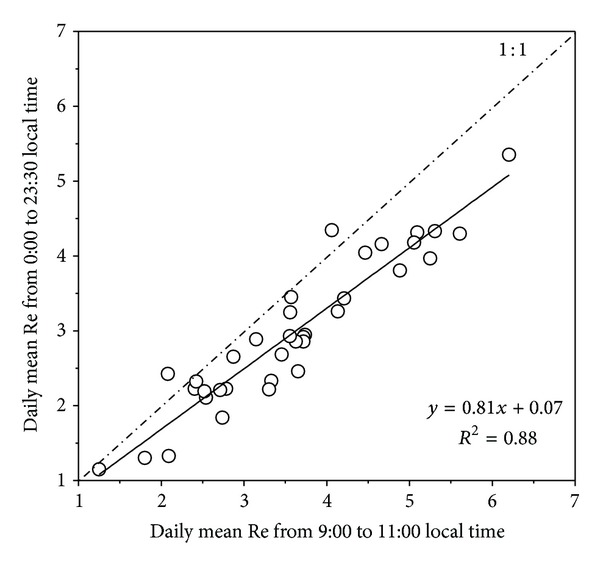
Relationships between the daily mean values of Re (*μ*moL m^−2^ s^−1^) calculated from 0:00–23:00 h local time and the Re estimated by 9:00–11:00 h a.m. local time; all the measurements for three chambers for all the sampling dates were pooled together, *n* = 36.

The maximum and minimum Re values of ecosystem respiration occurred at 12:00 to 16:00 h p.m. and 4:00 to 8:00 h a.m. local time, respectively (Figure 1), which corresponded well with the diurnal pattern of soil temperatures. Although the daily mean Re from 9:00 to 11:00 h a.m. local time was strongly correlated with that from 0:00 to 23:30 h (Figure 2), the calculated daily mean values of Re from 9:00 to 11:00 h a.m. local time were significantly higher than those from 0:00 to 23:00 h for both conditions with and without exclusion of wind effects (Table 1). Compared with daily mean values from 0:00 to 23:00 h, the Re values from 9:00 to 11:00 h a.m. local time were 23.90%/24.08% greater than the daily mean values before/after exclusion the effects of wind during the whole growing season (Table 1).

**Figure 3:**
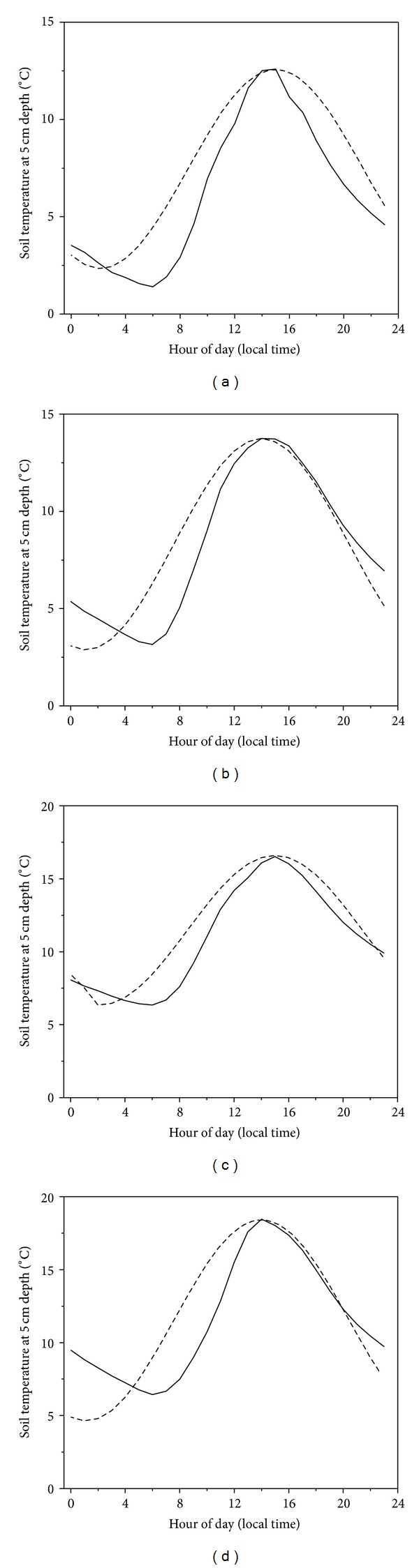
Comparison between 3-day daily mean diurnal soil temperature (solid line, 5 cm) and the corresponding sinusoidal temperature (dashed line) with the same daily range for May (a), June (b), July (c), and August (d).

Our results are consistent with Cao et al. [24] and Zhang et al. [25], whose results showed that diurnal variations of soil respiration on the alpine meadow presented significant single peak dynamics with the maximum value in 12:00–16:00 h p.m. local time and the minimum value in 4:00–8:00 h a.m. local time by using static chamber. However, our results are inconsistent with Tang et al. [26] and Li et al. [27], who have demonstrated that CO_2_ fluxes measured at 9:00–11:00 h a.m. local time were close to daily means in subtropical forest ecosystem in southern China and in cropland ecosystem on the Loess Plateau in northern China. Those correlations that existed in forest and cropland ecosystems were associated with relatively low range of soil temperature and flat daily curves of CO_2_ fluxes during daytime and nighttime [28]. In the present study, daily mean Re calculated from 9:00 to 11:00 h a.m. local time was significantly higher than 0:00 to 23:30 h local time, which might be caused by special climate conditions on the Qinghai-Tibetan Plateau. The solar radiation can penetrate thin atmosphere easily to heat land surface, the soil temperature increases quickly after sunrise; the upward land surface longwave radiation can also dissipate quickly due to thin atmosphere, and surface soil temperature decreases quickly in the afternoon (Figure 3). Stronger diurnal variation of Re was apparent in this alpine meadow ecosystem, it is possible that daily mean Re will be overestimated if it is represented by Re of any period during daytime. To improve the accuracy in estimation of carbon budget, hence, automated continuous all-day field observation for Re initiated here will provide more definitive studies [29, 30]. Compared with static chamber, automated soil CO_2_ flux system can measure wide temporal variability in ecosystem respiration with the range from half-hour to seasonal and even to interannual [31]. An alternative way is to calculate the ratios of instant Re from 9:00–11:00 a.m. local time to the daily mean Re and then to use these ratios to calculate daily mean Re [32].

## Figures and Tables

**Table 1:** Daily mean values of Re calculated from 9:00 to 11:00 h a.m. local time and from 0:00 to 23:00 h before and after exclusion of the effect of wind.

Date	Before exclusion of the effect of wind			After exclusion of the effect of wind		
	0:00–23:30	9:00–11:00	Overestimate (%)	0:00–23:30	9:00–11:00	Overestimation (%)
22-May	1.09	1.32	21.13	1.15	1.25	9.09
02-Jun	1.33	2.09	57.69	1.32	2.09	58.13
14-Jun	2.09	2.54	21.94	2.11	2.54	20.71
20-Jun	2.86	3.72	30.23	2.86	3.72	30.23
05-Jul	2.39	3.65	53.05	2.46	3.65	48.55
17-Jul	2.19	2.52	15.07	2.19	2.52	15.07
21-Jul	2.65	2.88	8.42	2.65	2.88	8.42
24-Jul	2.68	3.46	28.96	2.72	3.46	27.09
03-Aug	5.63	6.20	10.18	5.35	6.20	15.85
19-Aug	2.87	3.63	26.58	2.86	3.63	27.14
23-Aug	2.93	3.56	21.54	2.93	3.56	21.54
03-Sep	2.21	2.71	22.91	2.21	2.71	22.91

Mean	2.58	3.19	23.90	2.57	3.19	24.08
